# Dysphagia secondary to anterior cervical spine osteophytes

**DOI:** 10.11604/pamj.2014.17.36.3843

**Published:** 2014-01-20

**Authors:** Ali Akhaddar, Mohammed Zalagh

**Affiliations:** 1 Department of Neurosurgery, Avicenne Military Hospital, Marrakech, Morocco; 2 University of Mohammed V Souissi, Rabat, Morocco; 3 Department of Otorhinolaryngology, Moulay Ismail Military Hospital, Meknes, Morocco

**Keywords:** Dysphagia, cervical spine, osteophytes

## Image in medicine

A 40-year-old woman, previously healthy, presented with a 6-month history of foreign body sensation of the pharynx with recent progressive dysphagia when swallowing both solid and liquid foods. A lateral cervical radiograph showed severe anterior osteophytosis on C3-C4, which was confirmed on computed tomography scan (A, B and C). The posterior pharyngeal wall was compressed by the anterior spurs at the C3-C4 level. Anterior resection of the ventral spinal osteophytes was performed via an antero-lateral extrapharyngeal approach. After the operation, the dysphagia resolved. Cervical osteophytes are common but osteophytes causing dysphagia due to compression of pharynx and oesophagus are unusual. The most common aetiologies are diffuse idiopathic skeletal hyperostosis (Forestier's disease) and ankylosing spondylitis. Initial treatment includes diet modifications, non-steroidal anti-inflammatory and muscle relaxants medications. Osteophytectomy may be considered in certain patients where conservative management fails. Stabilization of the spine is not advocated.

**Figure 1:**
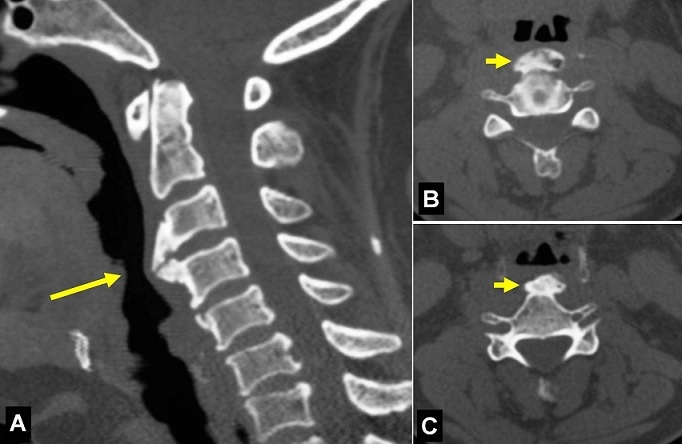
Cervical computed tomography scan (body windows) on sagittal (A) and axial (B and C) views revealing an important anterior cervical osteophytosis (arrows) at C3-C4 vertebral level causing compression of the upper airway and the pharynx

